# Development of an insertion device selectively operational as a helical/figure-8 undulator

**DOI:** 10.1107/S1600577522011997

**Published:** 2023-01-13

**Authors:** Takashi Tanaka, Takamitsu Seike, Akihiro Kagamihata, Hideki Aoyagi, Tomoya Kai, Mutsumi Sano, Sunao Takahashi, Masaki Oura

**Affiliations:** a RIKEN SPring-8 Center, Koto 1-1-1, Sayo, Hyogo 679-5148, Japan; b Japan Synchrotron Radiation Research Institute, Koto 1-1-1, Sayo, Hyogo 679-5198, Japan; Uppsala University, Sweden

**Keywords:** undulator, polarization, low heat load

## Abstract

An insertion device capable of switching the operation mode between helical and figure-8 undulators, and thus referred to as a helical-8 undulator, has been developed. It has the advantage that the on-axis heat load can be kept low regardless of the polarization state, even when a high *K* value is required to lower the fundamental photon energy. This is in contrast to conventional undulators in which the on-axis heat load tends to be significantly high to generate linearly polarized radiation with a high *K* value, and optical elements can be seriously damaged. The principle of operation, specification and light source performance of the developed helical-8 undulator are presented together with further options to enhance its capability.

## Abstract 2

An insertion device capable of switching the operation mode between helical and figure-8 undulators has been developed.

## 1. Introduction

Controlling the polarization state of synchrotron radiation (SR) is one of the most important subjects in the development of insertion devices (IDs). The most straightforward way is to employ an elliptically polarized undulator (EPU), whose magnetic field is given in a general form as

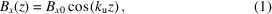




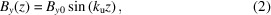

with *k*_u_ = 2π/λ_u_, where λ_u_ denotes the undulator period, *B*_*x*,*y*_ are the horizontal and vertical components of the magnetic field vector, *z* denotes the longitudinal coordinate, and *B*_*x*0,*y*0_ are the peak values of *B*_*x*,*y*_. The fundamental wavelength λ_1_ of undulator radiation is then defined as 

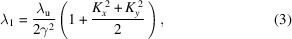

with 

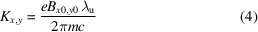

being the so-called (horizontal and vertical) deflection parameters or *K* values, where γ is the relative electron energy, *e* and *m* are the charge and rest mass of an electron, and *c* is the speed of light. It is well known that the spectrum of undulator radiation is quasi-monochromatic at the wavelengths of λ_1_/*n*, where *n* is a positive integer referred to as a harmonic order.

To control the polarization state flexibly, *B*_*x*0_ and *B*_*y*0_ should be independently tuned, with the magnet configuration satisfying the boundary condition of accelerator operation. For this purpose, a number of concepts have been proposed, and EPUs based on these concepts have been put into practical use (Yamamoto *et al.*, 1989 ▸; Elleaume, 1994 ▸; Sasaki *et al.*, 1993 ▸; Hara *et al.*, 1998 ▸; Bahrdt *et al.*, 2001 ▸; Schmidt & Calvi, 2018 ▸).

The EPU works as a linear undulator to generate linearly polarized radiation (LPR) when *B*_*x*0_ *B*_*y*0_ = 0 (*B*_*x*0_ = 0 or *B*_*y*0_ = 0), while it works as a helical undulator to generate circularly polarized radiation (CPR) when |*B*_*x*0_| = |*B*_*y*0_|; more specifically, it can generate horizontally polarized radiation (HPR), vertically polarized radiation (VPR), left-handed CPR, and right-handed CPR, by satisfying the conditions *B*_*x*0_ = 0, *B*_*y*0_ = 0, *B*_*x*0_ = *B*_*y*0_ and *B*_*x*0_ = − *B*_*y*0_, respectively.

Now let us turn to a well known issue regarding the operation of linear undulators: when the fundamental energy needs to be rather low, and thus relatively high *K* values are required (high-*K* condition), the radiation power coming from high-order harmonics becomes much higher than that from the fundamental radiation. This can potentially result in serious damage to optical elements such as mirrors and monochromators, and thus the heat load should be reduced by limiting the angular acceptance of optical elements usually by closing the aperture of the slit in the front-end section, which results in a significant loss of available photon flux. It should be stressed that this problem in the linear undulators is in contrast to the helical undulators, in which no high-order harmonics are contained in the radiation emitted on-axis, and thus the on-axis heat load is much lower.

The figure-8 undulator is an ID proposed to solve the above problem in the linear undulators under the high-*K* conditions (Tanaka & Kitamura, 1995 ▸), which generates a magnetic field given as 

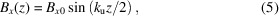




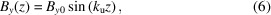

meaning that the period of the horizontal field is twice that of the vertical one; then the electron trajectory projected onto the transverse plane forms a ‘figure of eight’, and most of the radiation power coming from high-order harmonics diverges off-axis, keeping the linearly polarized fundamental radiation concentrated on-axis. As a result, the on-axis heat load is kept low even under the high-*K* conditions and is much lower than that of the conventional linear undulators. The figure-8 undulator has another advantage that the 0.5th-harmonic radiation, which is vertically polarized and is halved in energy, is available as well as the horizontally polarized fundamental radiation. Although the maximum photon flux is relatively lower than that of the fundamental radiation (depending on the ratio *K*_*x*_/*K*_*y*_), it offers a simple scheme to switch the polarization state; to be specific, HPR or VPR are available at the same photon energy just by tuning the gap of the undulator.

Figure-8 undulators have been constructed and installed at SPring-8 (Tanaka *et al.*, 1998 ▸) and ELETTRA (Diviacco *et al.*, 2002 ▸), as IDs for soft X-ray and vacuum ultraviolet beamlines, respectively, and have been successfully operated for more than two decades.

One disadvantage of the figure-8 undulators developed above is that CPR is not available. In 2011, we proposed a new undulator concept (Tanaka & Kitamura, 2011*a* ▸) to overcome this difficulty, which offers a scheme to switch the operation mode between helical/figure-8 undulators, and thus is referred to as a ‘helical-8 undulator’. Recently, we have built an insertion device based on this concept to generate LPR and CPR with a low on-axis heat load and installed at SPring-8. The purpose of this paper is to report on its design, specification and performance.

## 2. Principle of operation

**Figure 1:**
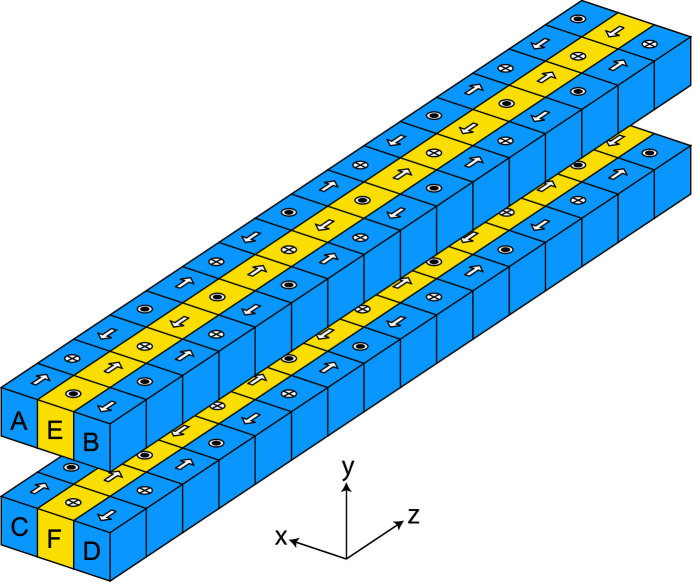
Schematic drawing of a helical undulator composed of six Halbach arrays. The definition of the coordinate system in this paper is shown for reference.

The magnet configuration of the helical-8 undulator is similar to that of a helical undulator composed of six Halbach undulator arrays as illustrated in Fig. 1 ▸, where the horizontal and vertical magnetic fields are independently generated by the side arrays (A–D) and central arrays (E, F), respectively. The helicity of CPR can be switched by moving the side arrays along the longitudinal axis by half a period. Helical undulators with this configuration have been constructed and installed at SPring-8 and several SR facilities before.

**Figure 2:**
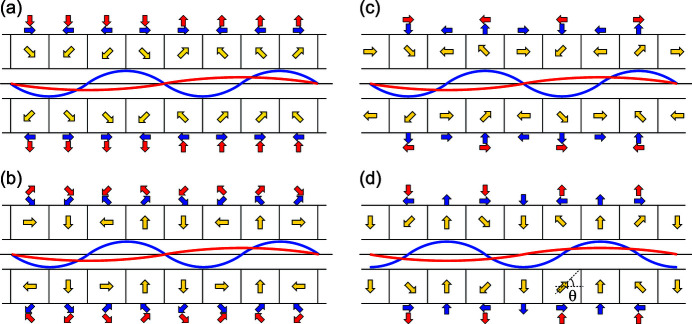
Four different undulator configurations to generate the composite period magnetic fields. Blue and red lines indicate the magnetic field distributions for the fundamental and double-period components, respectively.

The mechanism to switch the operation mode in the helical-8 undulator is based on a composite-period undulator (CPU) scheme, which was originally proposed to expand the wavelength tunability of X-ray free-electron lasers (Tanaka & Kitamura, 2011*b* ▸). The magnetic array in this scheme is modified from a conventional Halbach array to generate a magnetic field given as 

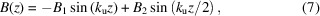

meaning that it is composed of two different periods, where *B*_1_ and *B*_2_ denote the peak values of the magnetic fields with the period of λ_u_ and 2λ_u_. In the following discussions, the former and latter are referred to as the fundamental and double-period components, respectively. Fig. 2 ▸(*a*) shows a schematic drawing of an undulator array to generate such a magnetic field. The yellow arrows indicate the magnetization vectors of individual magnet blocks, which are decomposed into two components indicated by blue and red arrows as shown nearby; the former and latter form the fundamental and double-period components, respectively.

Now let us consider the case when the top and bottom arrays are shifted by Δ*Z* along the longitudinal axis towards opposite directions; it is easy to show that the magnetic field reduces to 

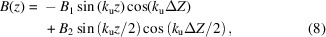

and then we have 

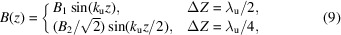

which means that the period of this undulator array can be switched between λ_u_ and 2λ_u_. As a result, the wavelength tunability can be significantly expanded compared with what is available with conventional undulators.

It should be noted that the configuration shown in Fig. 2 ▸(*a*) is not efficient in terms of attainable magnetic field strengths. As is well known, the peak field of the Halbach array is proportional to a geometrical factor given as 

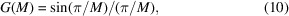

where *M* is the number of magnet blocks per period, and the magnetization vector of a specific magnet block is 2π/*M* rotated with respect to adjacent ones (Halbach, 1983 ▸). Although larger *M* results in a higher peak field, *M* = 4 is generally chosen, because *G*(4) ≃ 0.9 is usually acceptable. It is easy to understand that *M* is effectively 2 for both of the fundamental and double-period components in the configuration shown in Fig. 2 ▸(*a*). Recalling *G*(2) ≃ 0.63, the peak field attainable with this configuration is about 30% lower than what is usually available.

From a practical point of view, the peak field of the double-period component (*B*_2_) can be lower than that of the fundamental one (*B*_1_); to be specific, *B*_2_ can be as low as *B*_1_/2 to have the same *K* values. In such a case, we can enhance *B*_1_ at the expense of *B*_2_; an example is illustrated in Fig. 2 ▸(*b*), where *M* = 4 is attained in the fundamental component, but the Halbach condition is violated in the double-period component, which eventually results in lower *B*_2_. Fig. 2 ▸(*c*) is another example to proceed this idea further, where the double-period component is generated by tilting the vertically magnetized blocks in the original Halbach configuration. Fig. 2 ▸(*d*) shows an alternative configuration, where the phase relation between the fundamental and double-period components is shifted by 90°. In this case, we have 

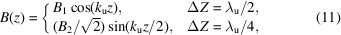

which are identical to equations (1) and (5), or the horizontal magnetic fields of the helical and figure-8 undulators. Now it is obvious that the undulator configuration shown in Fig. 1 ▸, with the side arrays A–D equipped with the CPU scheme explained in Fig. 2 ▸(*d*), works as a helical-8 undulator and can be selectively operated as a helical or a figure-8 undulator. It is worth noting that the relation between *B*_1_ and *B*_2_ depends on the angle θ indicated in Fig. 2 ▸(*d*), which is referred to as a CPU angle in the following discussions.

## 3. Specifications and performance evaluation

Based on the concept described in the preceding section, we developed a helical-8 undulator with λ_u_ of 120 mm and specifications summarized in Table 1 ▸, as an ID for the soft X-ray beamline BL17SU at SPring-8. The design and specification of the developed helical-8 undulator, which is referred to as HEU120 in this paper, are reported in this section, together with the light source performance experimentally demonstrated. Note that numerical studies presented in the following have been carried out using the numerical codes *RADIA* (Chubar *et al.*, 1998 ▸) and *SPECTRA* (Tanaka, 2021 ▸).

**Figure 3:**
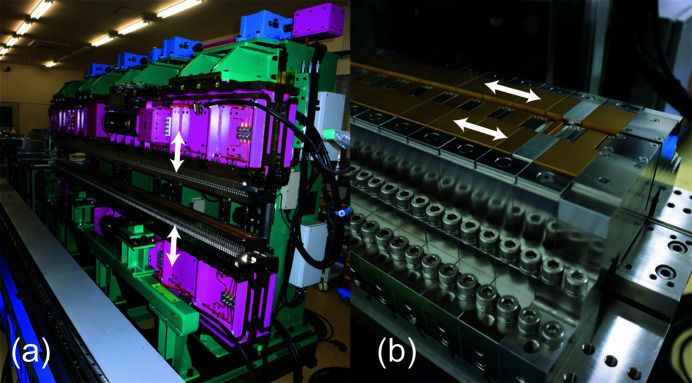
Photograph of HEU120 under construction: (*a*) mechanical frame and (*b*) magnetic arrays C, D and F.

Fig. 3 ▸(*a*) shows a photograph of the mechanical frame of HEU120 to hold the magnetic arrays and allow for the vertical motion to open and close the gap. Fig. 3 ▸(*b*) shows the bottom side of the magnetic arrays (C, D and F) mounted on a common girder of the mechanical frame; the side arrays (C and D) are equipped with a mechanical function to allow for the phase motion, or the longitudinal shift to switch the operation mode.

**Figure 4:**
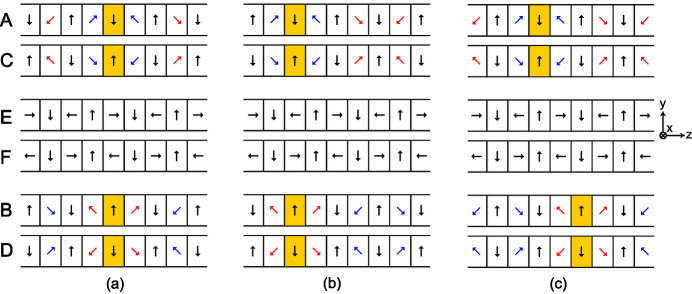
Operation modes of HEU120: (*a*) CW helical, (*b*) CCW helical and (*c*) figure-8 modes.

Phase conditions for respective operation modes are illustrated in Figs. 4 ▸(*a*)–4(*c*); for example, Fig. 4(*a*) shows the arrangement for the helical mode, where the side arrays generate the fundamental horizontal field without the double-period component. To facilitate the following discussions, let Δ*Z*_A,B,C,D_ be the longitudinal shifts of the side arrays A, B, C and D, with the origins (Δ*Z*_A,B,C,D_ = 0) being defined as the positions in this condition. By shifting the side arrays in the same direction by −λ_u_/2 (Δ*Z*_A,B,C,D_ = −60 mm) as shown in Fig. 4 ▸(*b*), the polarity of the horizontal field is flipped and the helicity of CPR is switched. For convenience, the former/latter modes are defined as the CW/CCW (clockwise/counter-clockwise) helical modes. Fig. 4 ▸(*c*) shows the arrangement for the figure-8 mode, where the (A, C) and (B, D) arrays are shifted by λ_u_/4 towards opposite directions (Δ*Z*_A,C_ = −Δ*Z*_B,D_ = −30 mm) to generate the double-period horizontal field without the fundamental component. Note that magnet blocks located in the center of the side arrays are painted yellow to clarify the positions of the side arrays in respective operation modes.

The light source performance of the helical-8 undulator is strongly dependent on the ratio *K*_*x*_/*K*_*y*_; let κ_*h*_ and κ_*l*_ be *K*_*x*_/*K*_*y*_ in the helical and figure-8 modes, respectively. It is obvious that the optimum condition in the helical mode is κ_*h*_ = 1, while that in the figure-8 mode depends on a number of conditions such as the acceptable heat load, tunable range, photon flux and degree of polarization.

**Figure 5:**
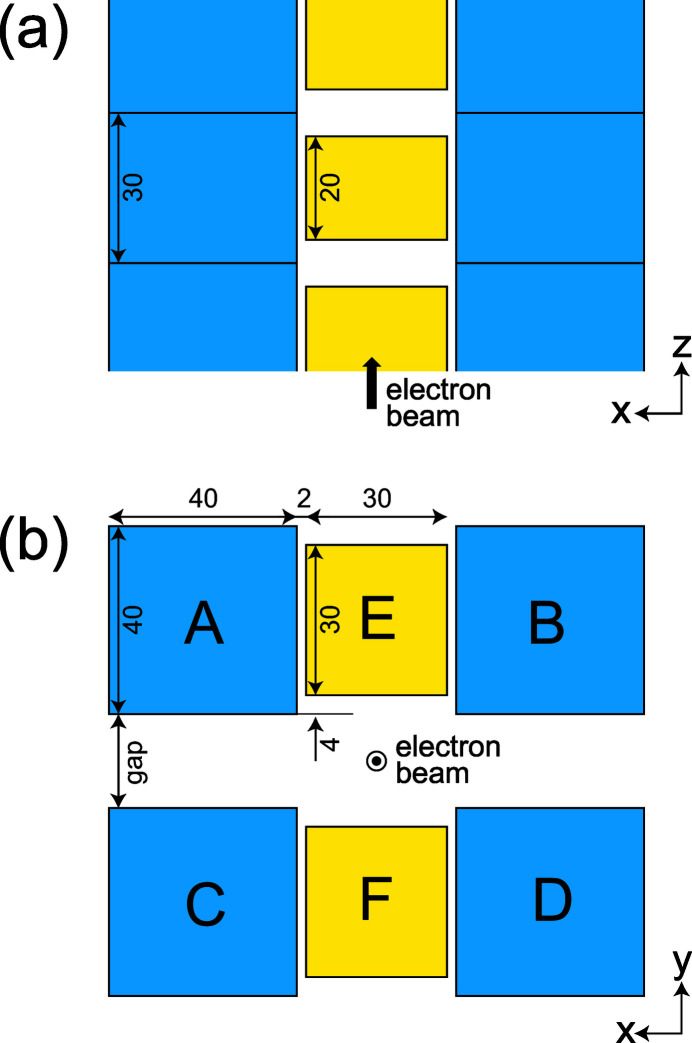
Dimensions (in mm) of magnet blocks in HEU120: (*a*) top view and (*b*) cross-sectional view.

Considering various boundary conditions and other factors besides the optimum conditions mentioned above, the dimensions of the magnet blocks have been determined as shown in Fig. 5 ▸, where we have three important points to be addressed. First, the magnet block length (20 mm) of the central array is slightly shorter than λ_u_/4 = 30 mm; the extra (10 mm-long) empty spaces are spent by mechanical clamps to fix the magnet blocks onto the common girder. Second, thanks to the relatively wide (30 mm) magnet block of the central array, the magnetic field is sufficiently uniform along the horizontal axis so that the dynamic multipole effect can be neglected. In other words, we do not have to apply special schemes such as multiwire coils to correct the dynamic multipole, as is often required for operation of EPUs. Third, the central array is slightly shifted up/downward with respect to the side array to weaken the vertical field and satisfy the condition κ_*h*_ ≃ 1.

Besides the dimensions of the magnet blocks described above, the CPU angle θ should be optimized according to the light source performance. As an example, we numerically evaluated the expected performance of HEU120 available in the figure-8 mode for three different values of θ, *i.e.* θ = 15°, 45° and 75°, which correspond to κ_*l*_ = 0.30, 0.96 and 1.45, respectively, The parameters used in the calculations are summarized in Table 2 ▸. Note that the fundamental photon energy is 500 eV, and the angular acceptance is four times the angular divergence of the photon beam.

**Figure 6:**
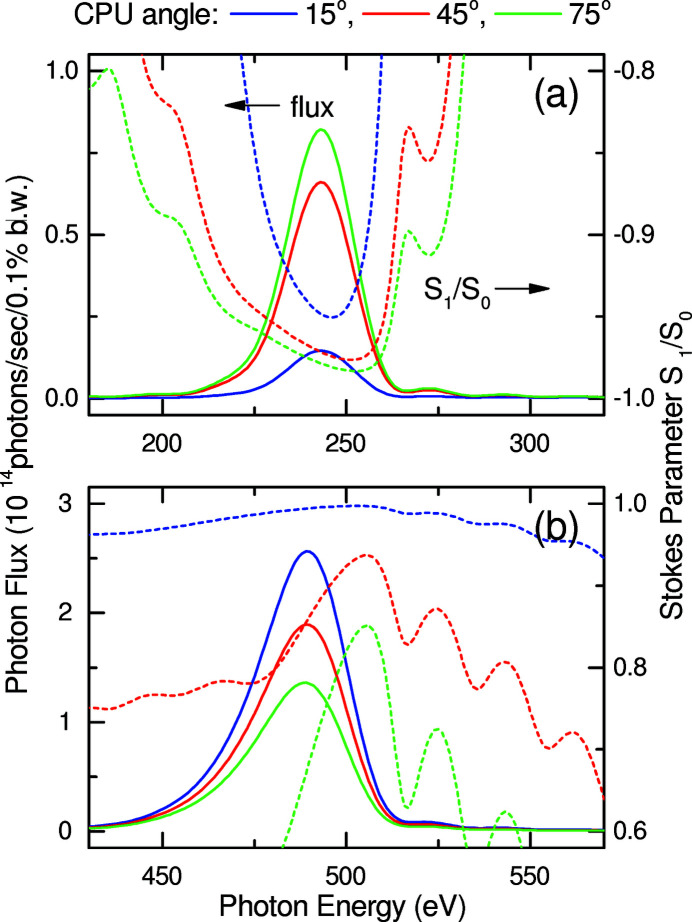
Comparison of light source performances between three different CPU angles: (*a*) 0.5th-harmonic and (*b*) fundamental radiation. Solid and dashed lines show the photon flux and Stokes parameter, respectively.

Figs. 6 ▸(*a*) and 6 ▸(*b*) show the light source performances available in the figure-8 mode with the 0.5th-harmonic and fundamental radiation, respectively, where the photon flux (solid lines) and Stokes parameter *S*_1_/*S*_0_ (dashed lines) representing the polarization property are plotted. Because of the large angular acceptance, the photon flux reaches maximum at the photon energy slightly detuned to the lower side, which is common to undulator radiation.

**Figure 7:**
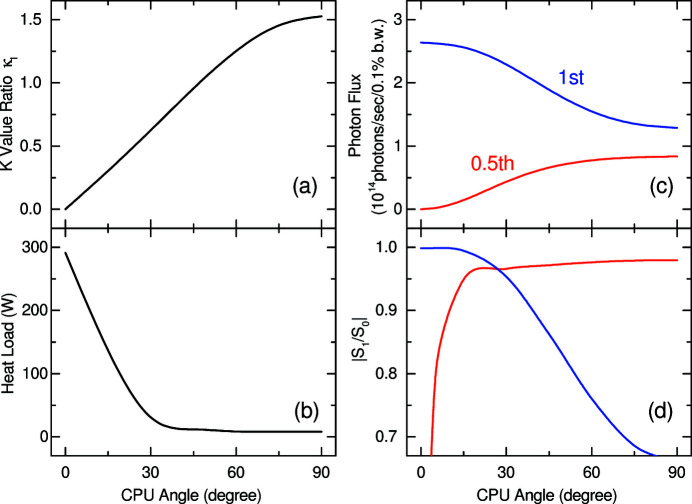
Light source performances plotted as a function of the CPU angle θ: (*a*) *K* value ratio κ_*l*_, (*b*) heat load, (*c*) photon flux and (*d*) polarization.

**Figure 8:**
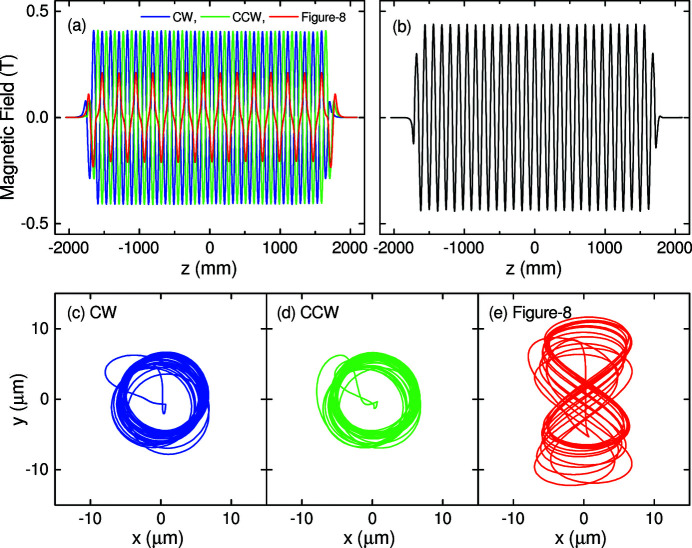
Results of the magnetic field measurement at the gap of 20 mm: (*a*) horizontal and (*b*) vertical field distributions, and (*c*–*e*) projections of electron trajectories (second field integral) evaluated for the three operation modes.

The above numerical process was repeated to evaluate the effect of the CPU angle on the light source performance, as summarized in Figs. 7 ▸(*a*)–7(*d*); the *K* value ratio κ_*l*_ (*a*), heat load of radiation (*b*), photon flux (*c*) and degree of polarization defined as |*S*_1_/*S*_0_| (*d*) are plotted as a function of the CPU angle. Note that the flux and polarization available at the detuned energy for the 0.5th-harmonic/fundamental radiation (VPR/HPR) are plotted in red/blue lines. As θ increases, the heat load is drastically reduced, which is the most important advantage of the figure-8 undulator. In addition, the photon flux of the 0.5th-harmonic radiation increases as well; however, the performance of the fundamental radiation degrades as θ. This means that we have to compromise in choosing the value of θ, and we have finally chosen θ = 45° focusing on the flux of the 0.5th-harmonic radiation and reduction of the heat load. From a technical point of view, this was a reasonable choice because we have already had several experiences of manufacturing magnet blocks with the 45° inclined easy axis of magnetization (Bizen *et al.*, 2018 ▸; Tanaka & Kagamihata, 2021 ▸).

Figs. 8 ▸(*a*) and 8 ▸(*b*) show the horizontal and vertical magnetic field distributions, respectively, measured at the minimum gap of 20 mm for the three operation modes. As shown in Fig. 8 ▸(*a*), the phase and period of the horizontal field change accordingly depending on the operation mode. Note that the vertical field does not depend on the operation mode and thus only one result is shown in Fig. 8 ▸(*b*). To evaluate the trajectories for the three operation modes, the field distributions shown in Figs. 8 ▸(*a*) and 8 ▸(*b*) are integrated twice; the results are plotted in Figs. 8 ▸(*c*)–8(*e*) in terms of the position of an 8 GeV electron projected on the transverse plane, where we find a typical trajectory specific to each operation mode, suggesting that HEU120 works as expected.

**Figure 9:**
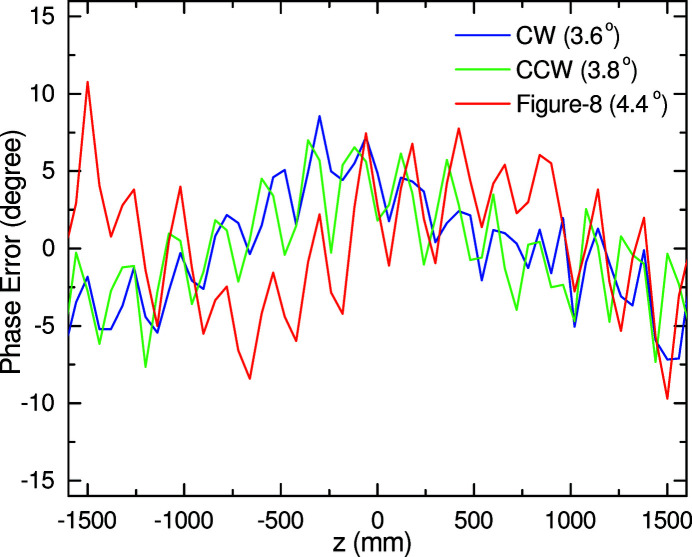
Phase errors evaluated from the measured field distributions for the three operation modes.

Fig. 9 ▸ shows the quality of the magnetic field of HEU120 in the three operation modes, where the phase errors evaluated from the field distributions shown in Figs. 8 ▸(*a*) and 8 ▸(*b*) are plotted as a function of the longitudinal coordinate, together with their RMS (root mean square) values. It is worth noting that only the 0.5th-harmonic and fundamental radiation are needed in HEU120 (applications using high-order harmonics are not supposed), and thus the RMS phase errors around 4° are sufficiently low; in other words, it is reasonable to say that the quality of HEU120 is close to ideal.

**Figure 10:**
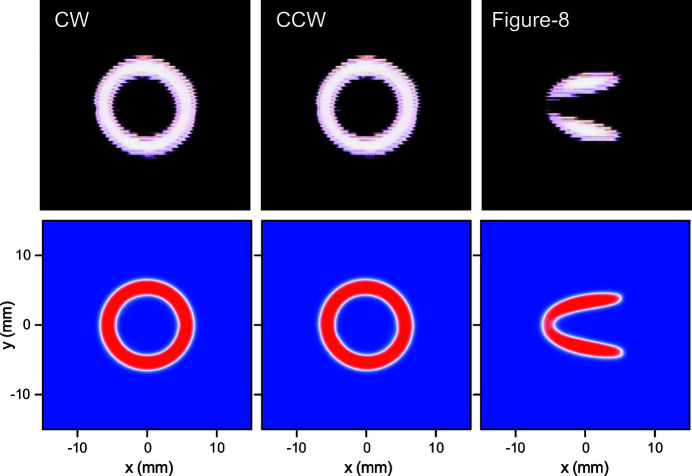
Top: profiles of SR measured by inserting a fluorescent screen. Bottom: radiation power density calculated with the measured field distributions.

Having verified the magnetic performance, HEU120 has been installed in the SPring-8 storage ring as an ID for BL17SU. To characterize the radiation and evaluate its performance quickly, we measured the spatial profiles of radiation in the three operation modes, by inserting an alumina fluorescent screen at the front-end section 19 m from the source; note that the beam current was reduced to 0.8 mA from the nominal one (100 mA), and a 1 mm-thick copper plate was inserted in front of the fluorescent screen to avoid saturation. The measurement results are shown in Fig. 10 ▸ (top), together with the spatial power density calculated with the measured field distributions (bottom). Due to several factors such as absorption by the copper plate and quantum efficiency of the fluorescent screen, the measured profile does not necessarily represent the radiation power density; even so, we find a relatively good agreement between the measured and calculated results.

## 4. Outlook

The experimental results shown in the preceding section strongly suggest that HEU120 has a sufficiently good performance as an ID to generate LPR and CPR with a low on-axis heat load. In other words, the helical-8 undulator concept, *i.e.* the six-array configuration equipped with the CPU scheme, works fine to switch the operation mode between the helical and figure-8 undulators. Nevertheless, we have to mention that HEU120 developed in this work has a disadvantage that the photon flux of the 0.5th-harmonic radiation (VPR) is rather lower than that of the fundamental radiation (HPR). This is not the case for the conventional EPUs, where the photon flux available for VPR is equivalent to that for HPR.

One idea to solve this problem is to apply the CPU scheme to the central arrays E and F as well as the side arrays, so that the vertical magnetic field can be switched according to equation (11) as well as the horizontal one. Then, another operation mode referred to as a ‘vertical figure-8 mode’ is available, in which the magnetic field is given as 

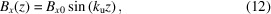




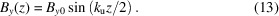

Because the above formulas are obtained by swapping the horizontal and vertical magnetic fields of the figure-8 undulator given in equations (5) and (6), the fundamental radiation is vertically polarized in this mode. Furthermore, ‘inclined’ linear polarization is available as reported in the previous paper (Tanaka & Kitamura, 2011*a* ▸), as well as the HPR and VPR. Note that the structure of the mechanical frame becomes more complicated to realize the above scheme, because all of the six arrays should be shifted along the longitudinal axis independently; it is worth mentioning, however, that such an ID to allow for the phase motion of six arrays has been constructed before (Tsuchiya *et al.*, 2016 ▸).

Introducing the vertical figure-8 mode mentioned above simultaneously brings an advantage in the design of the helical-8 undulator, in particular the choice of the CPU angle θ. As explained before using Fig. 7 ▸, we have two points to take care of: the photon flux of the 0.5th-harmonic radiation and reduction of the heat load. It is obvious that the former is no longer important if the vertical figure-8 mode is available. Thus, θ can be lower than 45° to improve the light source performance of the fundamental radiation, assuming that the resultant higher heat load is acceptable, and the technical issue in manufacturing the magnet blocks with an inclined easy axis can be solved. For example, θ = 30° is a reasonable choice as is obvious from Fig. 7 ▸.

Finally, let us compare the helical-8 undulator with another solution known as an ‘APPLE-Knot’ undulator (Zhang *et al.*, 2020 ▸). Although a higher degree of polarization is available in the linear polarization modes, the APPLE-Knot undulator cannot be operated as a purely helical undulator; instead, it generates a specially designed magnetic field to produce CPR. As a result, the photon flux of CPR available with the helical-8 undulator is expected to be much higher than that with the APPLE-Knot one.

## 5. Summary

We have presented the development of the helical-8 undulator, a new type of ID to generate LPR and CPR with a low on-axis heat load, by switching the operation mode between the helical and figure-8 ones. The light source performance experimentally demonstrated, together with the options to enhance its capability, definitely indicates that the helical-8 undulator can be a powerful candidate for IDs that need to control the polarization states under the high-*K* conditions. 

## Figures and Tables

**Table 1:** Specifications of HEU120 installed at SPring-8 BL17SU

Magnetic period	120 mm
Total length	3600 mm
CPU angle (θ)	45°
Minimum gap	20 mm
Maximum *K*_*y*_	4.76
Maximum *K*_*x*_	4.55 (helical)
	3.53 (figure-8)
Minimum photon energy	223 eV (helical)
	272 eV (figure-8)

**Table 2:** Parameters assumed in the numerical study to evaluate the light source performance of HEU120

Electron energy	8 GeV
Beam current	100 mA
Natural emittance	2.4 nm rad
Coupling constant	0.02%
Energy spread	0.0011
Betatron functions (*x*, *y*)	31.2 m, 5 m
Dispersion functions (*x*, *y*)	0.146 m, 0 m
Angular acceptance	0.1 mrad × 0.08 mrad
